# Folic acid deficiency optic neuropathy: A case report

**DOI:** 10.1186/1752-1947-2-299

**Published:** 2008-09-10

**Authors:** Punyanganie de Silva, Gerard Jayamanne, Robin Bolton

**Affiliations:** 1 Doncaster Royal Infirmary, Doncaster, DN2 5LT, UK; 2 Norfolk & Norwich University Hospital, Colney Lane, Norwich, NR4 7UY, UK

## Abstract

**Introduction:**

Nutritional optic neuropathies are uncommon and can be associated with gradual visual loss and optic atrophy or sudden vision loss and optic disc swelling.

**Case presentation:**

A 44-year-old woman presented with a 4-week history of progressive visual loss and was noted to have bilateral retrobulbar optic neuropathy. No other clinical abnormality was noted. Investigations revealed severe folate deficiency with normal vitamin B12 levels. Her alcohol and tobacco consumption was moderate and subsequent correction of folate levels with oral supplementation has led to improvement in her visual acuity.

**Conclusion:**

This case highlights an unusual presentation of folic acid deficiency that may present to the general physician.

## Background

Nutritional optic neuropathies are uncommon and can be associated with gradual visual loss and optic atrophy or sudden vision loss and optic disc swelling. The neuropathy is characterized by bilateral reduction in visual acuity. Patients may also complain of reduced colour vision and central or caecocentral scotomas may be noted on examination.

Folic acid deficient optic neuropathy has occasionally been reported in patients with a history of alcohol and/or tobacco consumption which does not need to be excessive. It is believed that alcohol and tobacco consumption contribute to poor appetite and hence poor nutritional status.

## Case presentation

A 44-year-old woman presented to A&E with a 2-week history of bilaterally progressive painless visual deterioration. She denied any other symptoms and had been prescribed reading glasses a month previously, which she found unhelpful. Her optician referred her to the ophthalmology clinic where she was seen 9 days later and it was noted that her vision had deteriorated further to 6/60 bilaterally. Bilateral central scotomas were present on confrontational testing with a red pin. Goldman visual fields looking at peripheral fields were normal. The optic discs appeared normal and were not pale. She was unable to see any of the Ishihara test plates for colour vision. In view of the rapid deterioration in her vision, she was referred to the medical team for further investigations.

Her past medical history was unremarkable and she consumed 14 U of alcohol(1.5 bottles ie 1125 ml of 12.5% wine) per week and 20 cigarettes per day. She was a housewife who denied any exposure to toxic substances or loss of weight but did admit to poor dietary intake. Her diet usually consisted of a cup of milk tea and a slice of toast with butter for breakfast. She usually drank 2–3 cups of tea or coffee during the day and would have a bag of potato crisps for lunch or skip this meal. Her evening meal would usually include some meat and potatoes but she admitted to having a limited intake of vegetables, particularly of the green leafy type and usually had these once or twice a month.

Clinical examination was normal apart from the previously noted visual abnormalities. Investigations revealed a folic acid level in the serum of 1.5 ng/ml (2.5–18 ng/ml), red cell folate 208 nmol/l (220–620 nmol/l), haemoglobin 14 g/dl (11.5–16 g/dl) with a mean corpuscular volume (MCV) of 122.8 fl (78–100 fl). Vitamin B_12 _levels were 354 nmol/l. It was interesting to note that she had had a history of low folate levels 3 years previously, but no further blood tests in the interim.

Thyroid function tests, endomysial and gliadin related antibodies were negative. An MRI brain scan revealed a tiny focus of high T2 signals in the left frontal lobe consistent with a benign perivascular space, but no evidence of demyelination. A diagnosis of folate deficient optic neuropathy was subsequently made and she was commenced on long-term oral folate supplementation of 5 mg/day. She was also advised to reduce her alcohol and tobacco consumption and a further ophthalmology follow-up arranged where it was noted 4 weeks later that her vision had improved to 6/36 bilaterally.

## Discussion

Folate deficient optic neuropathy is uncommon and early recognition and treatment are important to prevent persistent visual defects [1-3]. It is usually associated with starvation, malabsorption or excessive alcohol consumption. Dimness of vision is the outstanding symptom and it may initially be unilateral. It is a painless neuropathy. Alternative presenting symptoms are general loss of colour perception and dyschromatopsia. Other aspects of folate deficiency are axonal neuropathy, anaemia and encephalopathy.

The differential diagnoses of nutritional optic neuropathy include both toxic optic neuropathy – usually due to tobacco and alcohol, and Leber's hereditary optic neuropathy (LHON) which also presents as a gradual, painless deterioration in visual acuity [4]. It is therefore very important that a careful history is taken when evaluating patients with suspected optic neuropathy in order to exclude a nutritional deficiency. Details of the diet should be as precise as possible and a 24-hour recall of all food consumed is usually helpful. Other poor dietary habits such as erratic or infrequent meals, and low intake of green leafy vegetables should be actively sought. Whilst Leber's hereditary optic neuropathy is a mitochondrial disorder, folic acid deficient optic neuropathy is purely secondary to poor dietary intake. Men or women can be equally affected unlike LHON which is more common in young men. It is also important to remember that folate deficient optic neuropathy can occur in the absence of tobacco or alcohol consumption, unlike LHON which has a tendency for those who drink alcohol/consume tobacco to be more susceptible to optic disease [4]. Careful consideration to all these factors should therefore be given before making a definite diagnosis.

It is possible that serum folate levels may be normal in the presence of true folate deficiency as red cell folate levels are a more sensitive marker. Therefore, if a deficiency in folic acid is suspected, both serum and red cell folate levels should be analyzed.

Folate levels tend to be lower in women but it has been noted that men with low folate levels have a greater incidence of neuropathy than their female counterparts. In addition to markers of general nutritional status, MRI of the optic nerves and chiasm is indicated unless one is absolutely sure of the diagnosis. Formal visual field measurement is absolutely essential in the evaluation, and central or caecocentral scotomata with preservation of the peripheral field are characteristic visual field defects.

The anterior visual pathway is susceptible to damage from toxins or nutritional deficiency. The exact mechanism(s) by which nutritional deficits damage the optic nerve has not been elucidated but it is believed that the increased vascular supply of the optic nerve bundle increases its susceptibility to toxaemia. Folic acid is needed for the production of tetrahydrofolate, which is involved in detoxification of formate. Formate accumulation inhibits cytochrome oxidase thereby blocking mitochondrial oxidative phosphorylation [5]. Martin-Amat et al. reproduced optic disc swelling characterized by intra-axonal swelling and mitochondrial disruption in rhesus monkeys injected with toxic levels of formate [6]. Sadun noted that mitochondrial impairment in rats secondary to increased formate levels due to methanol injection subsequently led to decreased ATP production [7]. This subsequently impaired axon transport, preferentially involving less myelinated long fibres that were more prone to fire such as those found in the optic nerve and retinal nerve fibre layer.

It was also noted that those rats given a folate deficient diet were more severely affected than those which were not [7]. Golnik and Schaible [2] hypothesized that the folic acid deficient individual would be unable to detoxify endogenous formate subsequently resulting in formic acidaemia.

Vision can return to normal with vitamin supplementation and a balanced diet despite no modification of alcohol/tobacco consumption [1]. This is in contrast to alcohol related optic neuropathy and LHON where improvement post-treatment is variable [4,8]. Patients should be observed initially every 4–6 weeks and then, depending on their recovery, every 6–12 months. At each visit, the patient's visual acuity, colour vision, visual fields, pupils, and optic nerves should be assessed. Lack of compliance with therapy may lead to profound bilateral visual loss but never total blindness. Unless the loss of vision is already far advanced, the prospect for recovery or at least improvement is excellent, except for the most chronic cases. The rate of recovery varies from a few weeks to several months. Men are also more responsive to folate treatment than women [9].

Folic acid deficient optic neuropathy has a good prognosis if treatment is initiated in the first few months after the onset of symptoms. It has been noted that visual acuity tends to recover before colour vision and when recovery is complete, recurrences are unusual [9].

## Conclusion

In conclusion, in this case folate deficiency presented as visual abnormalities, and showed it is possible to have folate deficient optic neuropathy with no other clinical manifestation of folate deficient anaemia, and that folate deficiency can occur in the absence of excessive alcohol and tobacco consumption. This case is an unusual manifestation of nutritional deficiency which may present to the general physician, but when recognized promptly, can be corrected with vitamin supplementation.

## Consent

Written informed consent was obtained from the patient for publication of this case report. A copy of the written consent is available for review by the Editor-in-Chief of this journal.

## Competing interests

The authors declare that they have no competing interests.

## Authors' contributions

PdeS was involved in the care of the patient, drafting and writing the manuscript and literature review. RB treated the patient and critically revised the manuscript. GJ treated the patient and critically revised the manuscript. All authors read and approved the final manuscript.
